# Topical or oral antibiotics in childhood acute otitis media and ear discharge: a randomized controlled non-inferiority trial

**DOI:** 10.1093/fampra/cmae034

**Published:** 2024-06-24

**Authors:** Saskia Hullegie, Roger A M J Damoiseaux, Alastair D Hay, Nicolaas P A Zuithoff, Thijs M A van Dongen, Paul Little, Anne G M Schilder, Roderick P Venekamp

**Affiliations:** Julius Center for Health Sciences and Primary Care, University Medical Center Utrecht, Utrecht University, Utrecht, The Netherlands; Julius Center for Health Sciences and Primary Care, University Medical Center Utrecht, Utrecht University, Utrecht, The Netherlands; Centre for Academic Primary Care, School of Social and Community Medicine, University of Bristol, Bristol, United Kingdom; Julius Center for Health Sciences and Primary Care, University Medical Center Utrecht, Utrecht University, Utrecht, The Netherlands; General Practitioners Practice Tilburg-West, Tilburg, The Netherlands; Primary Care Research Centre, Primary Care Population Sciences and Medical Education Unit, Faculty of Medicine, University of Southampton, Aldermoor Health Centre, Southampton, United Kingdom; Julius Center for Health Sciences and Primary Care, University Medical Center Utrecht, Utrecht University, Utrecht, The Netherlands; National Institute for Health Research University College London Hospitals Biomedical Research Centre, London, United Kingdom; evidENT, Ear Institute, University College London, London, United Kingdom; Julius Center for Health Sciences and Primary Care, University Medical Center Utrecht, Utrecht University, Utrecht, The Netherlands

**Keywords:** acute otitis media, ear discharge, primary care, oral antibiotics, antibiotic-corticosteroid eardrops, treatment

## Abstract

**Background:**

Current guidance suggests oral antibiotics can be considered for children with acute otitis media (AOM) and ear discharge, but there is an absence of evidence regarding the relative effectiveness of antibiotic-corticosteroid eardrops.

**Aim:**

To establish whether antibiotic-corticosteroid eardrops are non-inferior to oral antibiotics in children with AOM and ear discharge.

**Design and setting:**

Open randomized controlled non-inferiority trial set in Dutch primary care.

**Methods:**

Children were randomized to hydrocortisone-bacitracin-colistin eardrops (five drops, three times per day in the discharging ear(s)) or amoxicillin suspension (50 mg per kilogram of body weight per day, divided over three doses administered orally) for 7 days. The primary outcome was the proportion of children with resolution of ear pain and fever at day 3.

**Results:**

Between December 2017 and March 2023, 58 of the planned 350 children were recruited due to slow accrual for various reasons. Children assigned to eardrops (*n* = 26) had lower resolution rates of ear pain and fever at 3 days compared to those receiving oral antibiotics (*n* = 31): 42% vs 65%; adjusted risk difference 20.3%, 95% confidence interval −5.3% to 41.9%), longer parent-reported ear discharge (6 vs 3 days; *P* = .04), and slightly higher mean ear pain scores (Likert scale 0–6) over days 1–3 (2.1 vs 1.4, *P* = .02), but received fewer oral antibiotic courses in 3months (11 for 25 children vs 33 for 30 children), and had less GI upset and rash (12% vs 32% and 8% vs 16%, respectively).

**Conclusion:**

Early termination stopped us from determining non-inferiority of antibiotic-corticosteroid eardrops. Our limited data, requiring confirmation, suggest that oral antibiotics may be more effective than antibiotic-corticosteroid eardrops in resolving symptoms and shortening the duration of ear discharge.

**Key messages**

- This trial compared topical versus oral antibiotics in children with AOMd.
- Due to early termination of the trial non-inferiority could not be determined. Thus, further research is required.

## Introduction

Of children with acute otitis media (AOM), 15%–20% present with acute ear discharge due to a spontaneous perforation of the eardrum [1, 2]. These children experience equal levels of ear pain, suffer from more frequent AOM recurrences and hearing problems, and benefit more from antibiotic treatment than those with AOM who do not present with ear discharge [1, 2]. Current guidelines suggest clinicians can consider oral antibiotics for children with AOM and ear discharge (AOMd) [3, 4], but this must always be balanced against the risk of side effects and the significant public health danger of antibiotic resistance [5]. We have previously shown that treatment with antibiotic-corticosteroid eardrops is superior to oral antibiotics in children with ventilation tubes presenting with acute ear discharge [6]. The question is whether this strategy is also effective in children without ventilation tubes who present with acute ear discharge as one of the symptoms of AOM [7], arguing that the spontaneous perforation of the eardrum would provide an entry for topical antibiotics to act in the middle ear. Such approach would contribute to antibiotic stewardship and avoid systemic antibiotic side effects. We, therefore, conducted a randomized controlled trial comparing treatment with oral antibiotics and antibiotic-corticosteroid eardrops in children presenting in primary care with AOMd.

## Method

From December 2017 to February 2023, an open, individually randomized controlled non-inferiority trial was conducted in 52 primary care practices in the region Utrecht, the Netherlands, including 225 general practitioners (GPs). The trial’s rationale and details of its design have been reported in detail elsewhere [8] and a more detailed method section including standardized outcomes’ assessment can be found in Supplementary File 1.

In short, children aged 6 months to 12 years presenting to their GP with AOMd in one or both ears and either ear pain or fever or both were eligible for trial participation. Trial participants were randomized to oral antibiotics (amoxicillin suspension) or antibiotic-corticosteroid eardrops (hydrocortisone-bacitracin-colistin eardrops) for 7 days. The primary outcome was the proportion of children free from ear pain (ear pain score 0 on a 0–6 Likert scale [9, 10]) and fever (body temperature (Supplementary File 1) lower than 38.0°C [4]) at day 3, i.e. 72 hours after randomization. Secondary outcomes are listed in Supplementary Box 1. All analyses were performed according to the intention-to-treat principle. Because of its importance in non-inferiority trials, per-protocol analysis was also performed for our primary outcome [11].

## Results

### Participants

Fifty-eight of a planned 350 children were included in the trial; 27 were assigned to antibiotic-corticosteroid eardrops and 31 to oral antibiotics (Supplementary Fig. 1). The primary outcome was assessed in 57 children (98%). Daily parental diaries were available for the same 57 children and weekly for 55 (95%) children. Treatment adherence was 88% in the eardrops group versus 97% in the oral antibiotics group. Baseline characteristics of participants were well-balanced between the groups (Supplementary Table 1). The median age of included children was 28 months and 40% was younger than 2 years. At the baseline visit, parents reported ear pain in 95% of participants, and the trial doctor measured a body temperature of ≥ 38.0°C in 30% and diagnosed bilateral AOM in 31%. Bilateral ear discharge was present in 7% (*n* = 2) of participants in the eardrops group versus 13% (*n* = 4) in the oral antibiotics group.

### Primary outcome

Of the children assigned to eardrops 42% were free from ear pain and fever at day 3 versus 65% of those assigned to oral antibiotics; adjusted absolute risk difference: 20.3%, 95% confidence interval (CI) −5.3% to 41.9% (Table 1). This exceeds the predefined non-inferiority margin of 15%.

**Table 1.** Primary and secondary outcomes

Primary outcome	Antibiotic-corticosteroid eardrops (*n* = 26)	Oral antibiotics (*n* = 31)	Antibiotic-corticosteroid eardrops vs. oral antibiotics		
			Relative risk (95% CI)	Risk difference, % (95% CI)	Adjusted^a^ risk difference, % (95% CI)
Children without ear pain and fever at day 3—*n* (%)	11 (42.3)	20 (64.5)	0.66 (0.39 to 1.10)	22.2 (−3.2 to 47.6)	20.31 (−5.3 to 41.9)
Secondary outcomes	Antibiotic-corticosteroid eardrops (*n* = 26)	Oral antibiotics (*n* = 31)	Effect size		
Scores			Mean differences (95% CI)		*P*-value^b^
Mean ear pain score over the first 3 days - (Likert Scale, ± SD)	2.1 (0.21)	1.4 (0.20)	0.67 (0.13 to 1.21)		.016
Mean body temperature over the first 3 days - °C (± SD)	37.2 (0.08)	36.9 (0.07)	0.32 (0.13 to 0.51)		.001
Proportions			Risk Ratios (95% CI)		*P*-value^c^
Children with at most mild ear pain at day 3 *(Likert Scale < 3)* - *n* (%)	22 (84.6)	27 (87.1)	0.97 (0.79 to 1.20)		1.000
Children with parent-reported ear discharge at day 3*—n* (%)	15 (57.7)	6 (19.4)	2.98 (1.35 to 6.57)		.003
Children with otoscopically confirmed ear discharge at 2 weeks*—n* (%)	1 (4.0)	3 (9.7)	0.41 (0.05 to 3.73)		.620
Children with MEE^d^ at 2 weeks*—n* (%)	22 (84.6)	26 (83.9)	1.01 (0.81 to 1.26)		1.000
Children with otoscopically confirmed eardrum perforation at 2 weeks*—n* (%)	2 (7.7)	4 (12.9)	0.60 (0.12 to 3.00)		.678
Duration of symptoms			Rate Ratios (95% CI)		*P*-value^e^
No. of days with ear pain during the first 2 weeks (Likert ≥ 1) mean (SD)	6 (3.3)	4 (2.4)	1.42 (1.05–1.93)		.025
No. of days with fever during the first 2 weeks—mean (SD)	2 (2.4)	1 (1.5)	1.99 (1.06–3.73)		.032
No. of days with parent-reported ear discharge at day 3—mean (SD)	3 (1.0)	3 (0.8)	1.18 (0.87–1.59)		.283
No. of days with parent-reported ear discharge during the first 2 weeks—mean (SD)	6 (3.8)	3 (2.3)	1.63 (1.17–2.27)		.004
No. of days with parent-reported ear discharge at 3 months—mean (SD)	8 (6.2)	5 (4.6)	1.60 (1.07–2.38)		.021
Time to resolution of total symptoms—mean (SD)^f^	5 (2.8)	4 (2.2)	1.36 (1.02–1.82)		.037
Other					
No. of AOM recurrences at 3 months (symptom-free period of ≥ 28 days—*n*^g^	0	2			
No. of AOM recurrences at 3 months (symptom-free period of ≥ 14 days) – *n*^g^	3	2			

^a^Adjusted for age (< 2 versus ≥ 2 years) and laterality (uni- versus bilateral AOM). ^b^Mean ear pain score and mean body temperature were analysed with a linear regression model, adjusted for: age (< 2 versus ≥ 2 years) and laterality (uni- versus bilateral AOM); ^c^Between-group differences in proportions were tested with Chi-Square tests and risk ratios were calculated; ^d^MEE: middle ear effusion; ^e^Between-group differences (means) were tested using negative binomial regression analyses; ^f^Time to resolution of total symptoms (time to all of pain, fever, discharge, being unwell, sleep disturbance, and distress/crying being rated 0 or 1 on the Likert scale; ^g^AOM recurrences has been defined as the occurrence of new AOM related symptoms after a symptom-free period of either 28 days or 14 days.

Per-protocol analysis showed a comparable result (risk difference: 18.7%, 95% CI −7.4 to 44.8).

### Secondary outcomes

Results for the secondary outcomes are summarized in Table 1. The proportion of children with mild ear pain at day 3, the proportion of children with otoscopically confirmed ear discharge, eardrum perforation, and MEE at 2 weeks were similar in both groups. The mean ear pain score over the first 3 days (Likert scale 0–6) was 2.1 (standard deviation [SD] 0.21) in the eardrops group versus 1.4 (SD 0.20) in the oral antibiotics group (*P* = .02).

**Figure 1.**
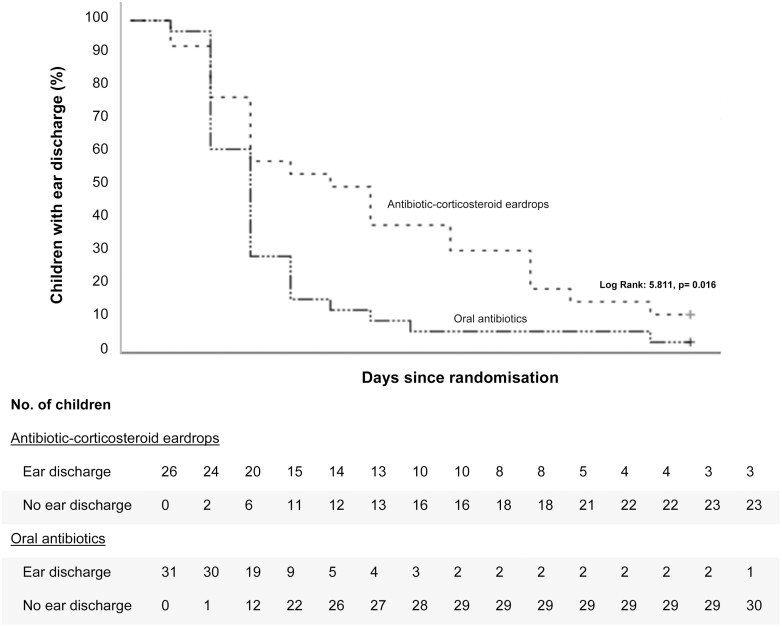
Kaplan–Meier Curve for the duration of ear discharge after randomization as reported by parents in a diary.

58% of children assigned to eardrops had parent-reported ear discharge at day 3 versus 19% of those assigned to oral antibiotics (*P* < .05). The mean duration of parent-reported ear discharge was 6 days (SD 3.8) in children receiving eardrops compared to 3 days (SD 2.3) for those receiving oral antibiotics (Kaplan–Meier curves, Fig. 1; Log Rank: 5.811, *P* = .02).

The mean time to resolution of total symptoms was 5 days (SD 2.8) in the eardrops group versus 4 days (SD 2.2) in the oral antibiotics group (*P* = .04). The total number of oral antibiotic courses in three months was 11 for 25 children in the eardrops group versus 33 for 30 children in the oral antibiotics group.

The OM-6-specific quality of life improved during follow-up in both groups (Supplementary Table 2).

### Adverse events

Discomfort during administration of the study medication was reported in 26% of children in the eardrops group versus 19% in the oral antibiotics group (Supplementary Table 3). For gastro-intestinal (GI) upset and body rash these percentages were 12% versus 32% and 8% versus 16%, respectively. No serious adverse events were reported during the 3 months of follow-up.

## Discussion

### Summary

Due to early termination of the trial non-inferiority of antibiotic-corticosteroid eardrops to oral antibiotics could not be determined in children with AOMd. In our small group of 58 children, we found that those assigned to eardrops had lower resolution rates of ear pain and fever at 3 days, longer parent-reported ear discharge, and slightly higher mean ear pain scores over days 1–3 compared to those receiving oral antibiotics, but received fewer oral antibiotic courses in three months and had less GI upset and rash.

### Strengths and limitations

This is the first report on the comparative effectiveness evidence of antibiotic-corticosteroid eardrops versus oral antibiotics in children with AOMd. The pragmatic design of the trial and high rate of data completeness support the applicability of its findings to routine daily practice.

Some limitations deserve further attention. Accrual to our trial was affected by a temporary closure due to study medication supply issues. When this was resolved the COVID-19 pandemic complicated trial recruitment and accrual did not recover after the pandemic restrictions were lifted. This phenomenon has affected many trials worldwide [12]. Further, our non-blinded design could potentially have introduced detection bias. However, detection bias is unlikely to have significantly impacted our findings since we compared two active treatments and—based on our parent panel input—parents do not have strong preferences for one over the other treatment. Also, a double-dummy design would have hampered the applicability of trial results to everyday practice.

We chose hydrocortisone-bacitracin-colistin eardrops because they are widely used in the Netherlands and France, do not contain a potentially ototoxic aminoglycoside, cover the most important pathogens involved in AOM, and have been proven effective in children with ventilation tubes who present with acute ear discharge [6]. They are however not available in many countries. Despite the absence of evidence, we believe that any combination of antibiotic-corticosteroid eardrops with a similar antimicrobial profile, like a quinolone-containing eardrop plus dexamethasone, would have yielded comparable results.

### Comparison with existing literature

We initiated this trial after establishing the superiority of antibiotic-corticosteroid eardrops over oral antibiotics in children with ventilation tubes who present with acute ear discharge [6]. Our current findings in a small sample of children without ventilation tubes who present with AOMd indicate that these findings cannot be extrapolated to this patient population. While there is a patent passage between the ear canal and the middle ear in children with ventilation tubes, the spontaneous eardrum perforation in children with AOMd may close too early to allow antibiotic-corticosteroid eardrops to completely resolve the middle ear inflammation.

During the preparation of our trial, we collaborated with the UK-based team developing the REST (Runny Ear Study, trial registry number ISRCTN12873692) addressing the same topic [13]. We harmonized design and outcomes to enable future meta-analysis. This trial however was also terminated early due to issues with its electronic health record system platform and no formal statistical analysis was performed on its sample of 22 children [13].

## Conclusions

We were unable to determine non-inferiority of antibiotic-corticosteroid eardrops to oral antibiotics, but our findings in a small group of children, requiring confirmation, suggest that oral antibiotics may be more effective in resolving symptoms and shortening the duration of ear discharge than antibiotic-corticosteroid eardrops in children with AOMd. That must be balanced against the findings that eardrops are associated with reasonable symptom control, fewer total oral antibiotic courses, and less systemic side effects in case there is non-inferiority. Since we were unable to demonstrate non-inferiority of antibiotic-corticosteroid eardrops to oral antibiotics in children with AOMd, current guidelines’ recommendation that clinicians can consider oral antibiotics in this group of children are not unreasonable but must be balanced against the major public health threat of antibiotic resistance.

## Supplementary Material

cmae034_suppl_Supplementary_Files_1_Figures_1_Tables_1-3

## Data Availability

The metadata is stored at the digital archive DataverseNL, https://dataverse.nl/dataset.xhtml?persistentId=doi:10.34894/J4KPP1.
