# Supplementary material for: Topical or oral antibiotics in childhood acute otitis media and ear discharge: a randomized controlled non-inferiority trial
Source: Fam Pract. 2024 Jun 24;41(5):857–61. doi: 10.1093/fampra/cmae034 (PMC11461153; doi:10.1093/fampra/cmae034)
Supplement: cmae034_suppl_Supplementary_Files_1_Figures_1_Tables_1-3 [file cmae034_suppl_supplementary_files_1_figures_1_tables_1-3.pdf]

Supplementary File

Supplementary 1 – detailed method section

Design and setting

From December 2017 to February 2023, an open, individually randomised controlled non-inferiority trial was conducted in 52 primary care practices in the region Utrecht, the Netherlands, including 225 general practitioners (GPs). Trial recruitment was put on hold from 8 August 2018 to November 2021 due to supply issues of one of the trial treatments (hydrocortisone-bacitracin-colistin (Bacicoline-B®) eardrops) and due to the COVID-19 pandemic.The trial's rationale and details of its design have been reported in detail elsewhere(1). The trial was reported according to the Consolidated Standards of Reporting Trials (CONSORT) guideline(2).

Participants

Children aged 6 months to 12 years presenting to their GP with AOMd in one or both ears and either ear pain or fever or both, were eligible for trial participation. AOMd is defined as the presence of acute-onset of otoscopically confirmed ear discharge together with other symptoms of an acute infection such as ear pain and/or fever, and/or irritability. The following children were excluded: ventilation tubes in place or a pre-existing perforation of the eardrum, systemically very unwell, having received antibiotics during the previous two weeks and having had an episode of AOM in the previous 28 days, known immunodeficiency, craniofacial malformation, Down's syndrome, previous ear surgery other than ventilation tubes, allergy to oral amoxicillin or hydrocortisone-bacitracin-colistin ear drops, and having already participated in the trial during a previous AOMd episode.

Randomisation

An independent data manager generated a computer-generated randomisation sequence with stratification according to age (<2 versus ≥2 years) and laterality (uni- versus bilateral AOM at baseline). After completing informed consent and baseline assessments, the trial doctor accessed a trial randomisation website for concealed study-group assignment. Assignment was balanced in a 1:1 ratio for the two study groups.

Procedures

Treatment groups

Children randomised to the oral antibiotic group were prescribed amoxicillin suspension, 50 mg per kilogram of body weight per day, divided over three doses administered orally for 7 days. Those randomised to eardrops were prescribed hydrocortisone-bacitracin-colistin eardrops, five drops, three times per day in the discharging ear(s) for 7 days. Parents were instructed to clean the ear of any visible ear discharge with a tissue and apply the drops while tilting their child's head to one side. During follow-up, any further treatment decisions were be up to the GPs' discretion.

Recruitment

GPs informed parents of potentially eligible children about the trial, took consent to share their contact details with the study team at the UMC Utrecht and provided a study information leaflet. Upon receipt of details, the trial doctor contacted parents by phone to provide detailed information about the study and scheduled a home visit on the same day for those who provisionally agreed to participate and whose child met the eligibility criteria.

Data collection

Baseline

At the home visit, the trial doctor obtained written informed consent from parents or guardians, checked the inclusion and exclusion criteria, collected demographic and disease-specific data and recorded otoscopic findings. Otoscopy was performed with a Welch Allyn MacroView Otoscope. Parents completed the Otitis media-6 (OM-6) questionnaire, an OM-specific QoL questionnaire on behalf of their child(3).

2-week follow-up

For the first 2 weeks of follow-up, parents kept a daily diary (paper or online) of AOM-related symptoms, treatment adherence, adverse events and complications of AOM for 2 weeks. Parents measured their child’s temperature two times per day (morning and evening) with a tympanic membrane thermometer in the unaffected ear. In children aged below 2 years and in those with bilateral ear discharge, body temperature was measured rectally. To standardise measurements, a study thermometer was provided to parents of participating children.
At 2 weeks, the trial doctor scheduled a follow-up home visit, where they verified the diary data and recorded otoscopy and tympanometry findings. Tympanometry was performed with a Interacoustics M10 tympanometer. A diagnosis of OME in one or both ears was based upon MOMES diagnostic algorithm which combines tympanometry and otoscopy findings(4). Parents completed the OM-specific QoL (OM-6) questionnaire (4,5).

3-month follow-up

After 2 weeks, parents kept a weekly diary (paper or online) recording AOM recurrence, GP consultations, medication use, hospital admissions, and societal costs for AOM for 3 months. At 3 months parents completed the OM-6 questionnaire, returned paper versions of the weekly diary and the questionnaire to the study team by mail; online versions were entered directly in the online database (Research Online).

Outcomes

The primary outcome was the proportion of children free from ear pain (ear pain score 0 on a 0-6 Likert scale(5,6)) and fever (body temperature lower than 38.0°C (7)) at day 3, i.e. 72 hours after randomisation. Secondary outcomes are presented in Supplementary Box 1.

Patient Involvement

We have established a parental panel. This parent panel was involved throughout all critical stages of the trial, including design and conduct of the research through regular parent panel meetings.

Sample size calculation

The clinically acceptable non-inferiority margin was set at 15%; that is, 50% of a 30% difference between oral antibiotics and placebo or no treatment as observed in earlier trials.(8,9). To demonstrate that the upper limit of a two-sided 95% confidence interval (CI) of the difference in treatment effect for the primary outcome does not exceed the predefined non-inferiority margin of 15% with 80% power, a minimum of 159 children per group was needed. To allow for 10% attrition, we aimed to randomise 350 children.

Statistical analysis

All analyses were performed according to the intention-to-treat (ITT) principle. Because of its importance in non-inferiority trials, per-protocol analysis was also performed for our primary outcome (8). Descriptive statistics were used to describe the baseline characteristics trial population. The primary outcome was analysed with a logistic regression model, to reduce small sample size bias, the model was estimated with Firth's correction. In addition to treatment group assignment, age and laterality were included as potential confounders. Risk differences (RDs) were derived from the regression model using the method described by Austin: bootstrapping techniques were performed to calculate accompanying 95% confidence intervals (CIs)(10). In sensitivity analysis, we assessed whether results differed when defining absence of fever for the primary outcome as parental fever score 0 or 1 (on the 0-6 Likert scale) at day 3 instead of the child's body temperature recordings as specified above.

For the dichotomous secondary outcomes, crude relative risks (RRs) and RDs with 95% CIs were calculated. The mean ear pain score and the mean fever score were analysed with a linear regression model. A residual covariance (i.e. GEE type) matrix was included to adjust for repeated measurements over time(11). In these analyses adjustments for time, age, laterality and baseline pain score were made. The validity of the model assumptions (i.e. homoscedasticity and normality) was evaluated by assessing residuals. Differences in dichotomous secondary outcomes were analysed with Chi-square tests and differences in duration of symptom outcomes were analysed using negative binomial regression. Kaplan-Meier curves were plotted for the duration of ear discharge and log-rank tests were used to test between group differences. All statistical analyses were performed with IBM SPSS Statistics (version 27.0), RStudio (version 2023.06.0) and with SAS (version 9.4).

References

1. Hullegie S, Venekamp RP, Van Dongen TMA, Mulder S, Van Schaik W, Ardine De Wit G, et al. Topical or oral antibiotics for children with acute otitis media presenting with ear discharge: study protocol of a randomised controlled non-inferiority trial. BMJ Open 2021;11:52128.
2. Schulz KF, Altman DG, Moher D, CONSORT Group. CONSORT 2010 Statement: updated guidelines for reporting parallel group randomized trials. Obstetrics & Gynecology 2010 May;115(5):1063–70.
3. Rosenfeld RM, Goldsmith AJ, Tetlus L, Balzano A. Quality of life for children with otitis media. Arch Otolaryngol Head Neck Surg 1997 Oct;123(10):1049–54.
4. Engel J, Anteunis L, Volovics A, Hendriks J, Marres E. Prevalence rates of otitis media with effusion from 0 to 2 years of age: healthy-born versus high-risk-born infants. Int J Pediatr Otorhinolaryngol 1999 Mar;47(3):243–51.
5. Watson L, Little P, Moore M, Warner G, Williamson I. Validation study of a diary for use in acute lower respiratory tract infection. Fam Pract 2001 Oct;18(5):553–4.
6. Little P, Gould C, Williamson I, Moore M, Warner G, Dunleavey J. Pragmatic randomised controlled trial of two prescribing strategies for childhood acute otitis media. BMJ 2001;322(7282).
7. Damoiseaux RAMJ, Venekamp RP, Eekhof J, Bennebroek Gravenhorst FM, Schoch AG, Burgers J, Bouma M, Wittenberg J. NHG-Standaard Otitis media acuta bij kinderen (derde herziening). Huisarts & Wetenschap 2014;(57):648
8. Wangge G, Klungel OH, Roes KCB, de Boer A, Hoes AW, Knol MJ. Interpretation and inference in noninferiority randomized controlled trials in drug research. Clin Pharmacol Ther 2010 Sep 28;88(3):420–3.
9. U.S. Department of Health and Human Services Food and Drug Administration, (CDER), Center for Drug Evaluation and Research (CBER) C for BE and R. Non-Inferiority Clinical Trials to Establish Effectiveness. Guidance for Industry. 2016.
10. Austin PC. Absolute risk reductions, relative risks, relative risk reductions, and numbers needed to treat can be obtained from a logistic regression model. J Clin Epidemiol 2010 Jan;63(1):2–6.
11. Fitzmaurice GM, Laird NM, Ware JH. Applied Longitudinal Analysis. Wiley; 2014.

Supplementary Table 1. Baseline characteristics of trial participants

Characteristic	Antibiotic-corticosteroid eardrops (n=27)	Oral antibiotics (n=31)	All children (n= 58)
Age			
months, median [IQR]	28 [13 to 64]	28 [17 to 52]	28 [15 to 55]
< 2 year, n (%)	11 (40.7)	12 (38.7)	23 (39.7)
Sex, male, n (%)	13 (48.1)	14 (45.2)	27 (46.6)
Medical history, n (%)			
Previous AOMd[a]	14 (51.9)	16 (51.6)	30 (51.7)
Recurrent URTI (>6 in 1 year)	15 (55.6)	17 (54.8)	32 (55.2)
Atopic constitution	9 (33.3)	11 (35.5)	20 (34.5)
Symptoms prior to enrolment			
Duration of AOMd[b] – days, median	2 [1 to 3.5]	3 [1-5]	3 [1 to 4]
Symptoms at baseline			
Ear pain, n (%)	26 (96.3)	29 (93.5)	55 (94.8)
Fever (≥38°C)[c], n (%)	8 (29.6)	9 (29.0)	17 (29.8)
Parent/GP reported fever[d], n (%)	16 (59.3)	17 (54.8)	33 (56.9)
Physical examination, n (%)			
Temperature, °C, mean ±SD	37.6 ±0.84	37.7 ±0.95	37.6 ±0.89
Parent/GP reported temperature[d], °C, mean ±SD	38.1 ±0.98	38.1 ±0.89	38.1 ±0.93
Bilateral AOM	9 (33.3)	9 (29.0)	18 (31.0)
Bilateral AOMd	2 (7.4)	4 (12.9)	6 (10.3)
Risk factors, n (%)			
Pneumococcal vaccination	25 (92.6)	30 (96.8)	55 (94.8)
Household smoking	0 (0)	4 (12.9)	4 (6.9)

[a]Previous AOMd: a previous episode of acute otitis media and ear discharge in the medical history. [b]Duration of AOMd (3 missings; group 1: n = 26; group 2: n=20; total: n=55). [c]One missing baseline temperature group 2 (n=30). [d]Fever/temperature**:** based on the combination of the baseline visit temperature and the temperature measured by either the GP or the parents within the 24 hours preceding the baseline visit.

Supplementary Table 2. Disease specific quality of life assessed with the otitis media-6 questionnaire at baseline, at 2 weeks and 3 months follow up.

Questionnaire	Range of scores°	Baseline (n=57)		Week 2 (n=56)		Month 3 (n=48)		Change score*		Difference in change score±	Change score *		Difference in change score ±
		Eardrops mean (SD)	Oral Abx mean (SD)	Eardrops mean (SD)	Oral Abx mean (SD)	Eardrops mean (SD)	Oral Abx mean (SD)	Eardrops Δ T0-T2 (SD)	Oral Abx Δ T0-T2 (SD)	Eardrops vs oral Abx Δ T0-T2 (p)	Eardrops Δ T2-T3 (SD)	Oral Abx Δ T2-T3 (SD	Eardrops vs oral Abx Δ T2-T3 (p)
Physical suffering	1 to 7	5.2 (1.68)	5.9 (1.26)	5.1 (1.68)	4.6 (1.63)	1.9 (1.29)	2.2 (1.44)	0.1 (1.68)	1.3 (1.39)	-1.19 (p=0.006)	3.4 (1.66)	2.6 (2.39)	0.76 (p =0.207)
Hearing loss	1 to 7	3.2 (1.83)	3.0 (2.05)	3.1 (1.90)	3.7 (1.8)	2.0 (1.40)	2.1 (1.37)	0.0 (1.71)	-0.7 (1.69)	0.71 (p = 0.127)	1.1 (1.52)	1.7 (1.69)	-0.57 (p= 0.234)
Speech impairment	1 to 7	2.1 (1.23)	1.8 ((1.20)	2.2 (1.41)	2.0 (1.13)	1.7 (1.34)	1.6 (0.69)	-0.2 (0.82)	-0.2 (1.43)	0.07 (p = 0.831)	0.5 (1.07)	0.6 (0.97)	-0.08 (p = 0.793)
Emotional distress	1 to 7	4.8 (1.77)	5.4 (1.23)	4.3 (1.67)	4.3 (1.3)	1.9 (0.97)	1.9 (1.03)	0.5 (1.6)	1.1 (1.14)	-0.63 (p= 0.097)	2.4 (1.64)	2.4 (1.72)	-0.01 (p = 0.988)
Activity limitations	1 to 7	4.6 (1.79)	4.7 (1.42)	4.1 (1.62)	3.9 (1.3)	1.9 (1.09)	1.9 (0.93)	0.5 (1.42)	0.8 (1.42)	-0.33 (p= 0.385)	2.3 (1.69)	2.2 (1.73)	0.15 (p = 0.764)
Caregivers concerns	1 to 7	4.7 (1.42)	4.7 (1.37)	4.1 (1.3)	3.8 (1.4)	2.1 (1.45)	2.1 (1.20)	0.4 (1.29)	0.9 (1.54)	-0.55 (p = 0.149)	2.1 (1.59)	1.8 (1.92)	0.29 (p = 0.581)
Mean symptom score	1 to 7	4.1 (1.24)	4.3 (0.95)	3.9 (1.33)	3.7 (1.11)	1.9 (1.11)	2.0 (0.99)	0.19 (0.98)	0.54 (0.91)	-0.36 (p = 0.164)	2.0 (1.22)	1.9 (1.47)	0.11 (p= 0.773)
Total score	6 to 42	24.3 (7.43)	25.5 (5.68)	22.8 (7.54)	22.2 (6.67)	11.4 (6.68)	11.7 (5.96)	1.5 (5.50)	3.3 (5.49)	-1.73 (p = 0.246)	11.6 (7.63)	11.2 (8.80)	0.42 (p = 0.864)
Visual analog score	0 to 10	4.4 (2.21)	4.4 (1.89)	4.8 (1.55)	5.1 (1.92)	7.8 (2.09)	7.8 (2.09)	0.4 (2.32)	0.7 (1.92)	-0.32 (p = 0.585)	3.0 (2.56)	3.8 (2.23)	-0.78 (p = 0.283)

Oral Abx; oral antibiotics ;°1-7: higher scores indicating more of a problem *Change score between time points, a positive value indicates clinical improvement; a negative value, deterioration. A score < 0.5 indicates trivial change; 0.5 - 0.9 small change, 1.0 - 1.4 moderate change; and > 1.5 large change; ± using independent t-test

Supplementary Table 3. Treatment-related and serious adverse events

	Antibiotic-corticosteroid eardrops (n=26)	Oral antibiotics (n=31)
**Adverse event within 2 weeks**	n (%)	n (%)
Local discomfort during administration	16 (61.5)	6 (19.4)
Difficulties during administration	15 (57.7)	3 (9.7)
Gastrointestinal discomfort	3 (11.5)	10 (32.3)
Rash - local (ear)	5 (19.2)	5 (16.1)
Rash - body	2 (7.7)	5 (16.1)
Dizziness	1 (3.8)	0 (0)
**Serious adverse events**	0 (0)	0 (0)

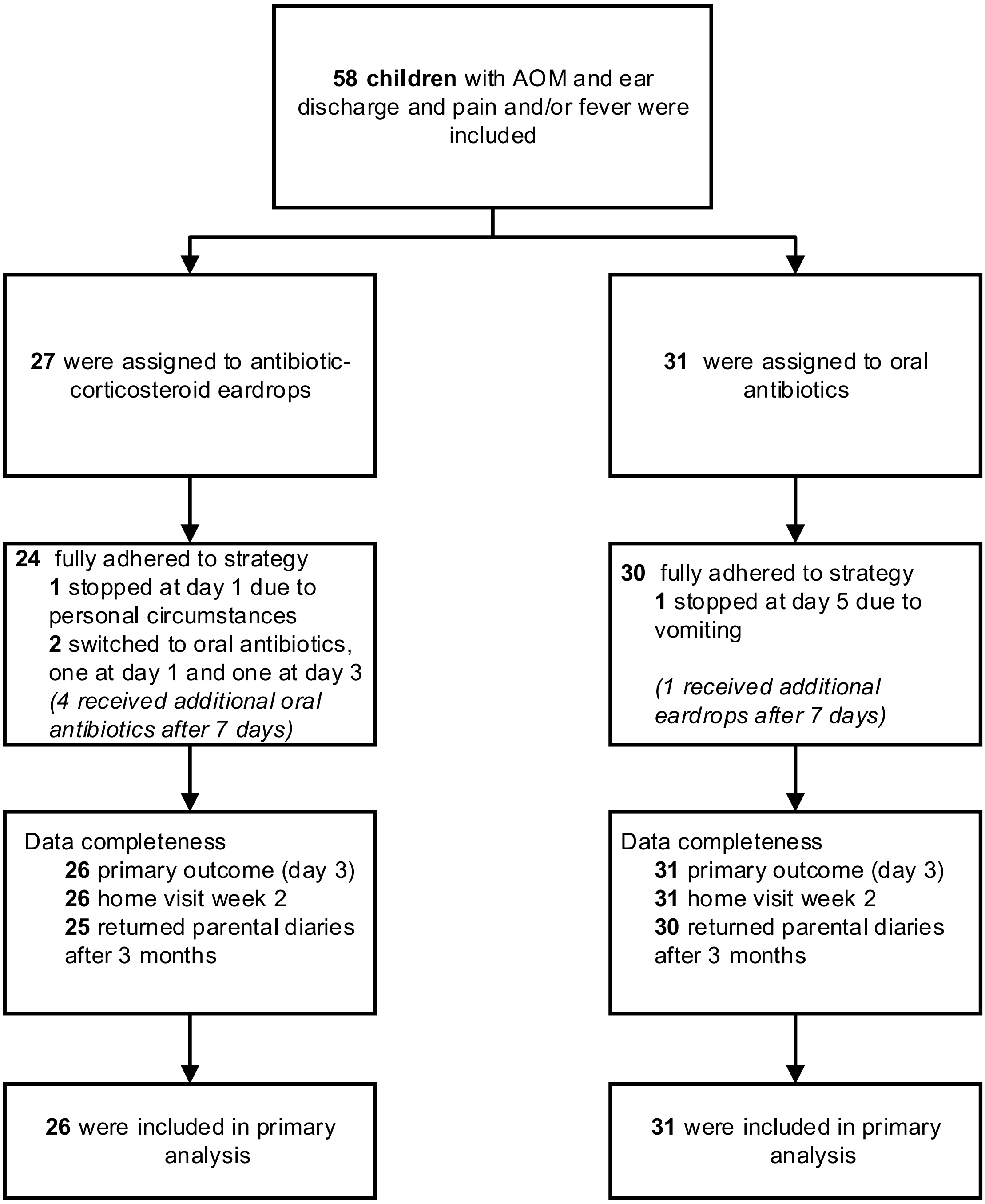

Supplementary Figure 1. Flowchart

Supplementary Box 1. Secondary outcomes

Ear pain	○ the mean ear pain score over the first 3 days;<br>○ the proportion of children with at most mild ear pain at day 3 (score less than 3 on the 0-6 Likert scale);<br>○ the number of days with ear pain score 1 or higher on the 0-6 Likert scale during the first 2 weeks.
Fever	○ the mean temperature over the first 3 days<br>○ the number of days with fever (at least one recording of a body temperature of 38.0°C or higher per day) during the first 2 weeks.
Ear discharge	○ the proportion of children with parent-reported ear discharge at day 3;<br>○ the number of days with parent-reported ear discharge during the first 2 weeks;<br>○ the proportion of children with otoscopically confirmed ear discharge at 2 weeks;<br>○ the number of days with parent-reported ear discharge at 3 months.
Time to resolution of total symptoms	○ time to all of pain, fever, ear discharge, unwell, disturbed sleep, and distress/crying being rated 0 or 1 on the Likert scale for two consecutive days.
Eardrum perforation	○ the proportion of children with an eardrum perforation at 2 weeks based on combined otoscopy and tympanometry findings.
OME	○ the proportion of children with OME (using the MOMES algorithm) in one or both ears at 2 weeks.
Antibiotic consumption	○ the total number of oral and ototopical antibiotics used during the first 2 weeks and at 3 months follow-up.
Adverse events	○ the adverse events during the first 2 weeks.
OM-specific quality of life	○ the OM specific quality of life assessed using the parent-reported OM-6 questionnaire at baseline, 2 weeks and 3 months.
AOM recurrences	○ the number of AOM recurrences during 3 months follow-up.
